# 

**Published:** 1894-03

**Authors:** 


### CONTENTS:

ORIGINAL ARTICLES: PAGE

The Anatomy of the Large American Fluke (*Fasciola Magna*), and a Comparison with other Species of the Genus Fasciola, S. St. By Charles Wardell Stiles, Ph.D. 161
Homeopathy a· Applied to Veterinary Med'cine. By F. B. Carlton, M.D.V......... 179
Prevention of Tuberculosis. By John M. Parker, D.V.S......................... 182
Notes on Kangaroos. By A. W. Whitehouse........................................ 187
General Remarks on Equine Ovariotomy. By James A. Waugh, V.S............... 195
Ovariotomy. By S. E. Willyoung................................................ 197
On the Emasculating Bot-fly...................................................... 200
Tuberculosis: its Relation to Long-continued In-breeding and Forced Lactation for High Milk, Butter and Cheese Testing. By A. S. Heath, M.D.................. 204
Problems in the Study of Horse-shoeing. By Williamson Bryden, V.S.............. 206
Tuberculosis. By Williamson Bryden, V.S.......................................... 207
Correction. T. J. Turner........................................................... 208

SELECTIONS:

Recent Researches on Tetanus. By R. T. Hewlett, M. D.......................... 209
Vomition in the Horse—Ejection of Ascarides. By J. B. Wolstenholme, F.R.C.V.S. 214
Prolonged Pregnancy. By W. T. Monsarrat.......................................... 216

EDITORIAL:

Army Veterinary Bill.................................................................. 217

COMMUNICATION:

Ohio Veterinary College................................................................ 218

SOCIETY PROCEEDINGS:

Veterinary Medical Association of New York County.............................. 221
Veterinary Medical Association of Oneida County................................. 222
Ohio State Veterinary Medical Association......................................... 223

